# Flexural Properties of Textile-Reinforced Concrete with Nonorthogonal Grids

**DOI:** 10.3390/polym14235185

**Published:** 2022-11-28

**Authors:** Tianqi Zhang, Boxin Wang, Xinyu Lu, Jiahuan Guo

**Affiliations:** College of Construction Engineering, Jilin University, Changchun 130061, China

**Keywords:** basalt textile-reinforced concrete, nonorthogonal grids, multi-crack failure, diagonal layout, four-point bending test

## Abstract

Textile-reinforced concrete (TRC) is a cement-based composite material that uses textile as a reinforcement material. The weft-direction fiber bundles in the traditional orthogonally arranged warp–weft textile hardly bear force, and its bonding strength with the weft fiber bundle is not ideal. Under the action of force, a small included angle between the stressed fiber bundle and the stressed direction can effectively increase the anchoring effect of their fibers in the matrix, resulting in higher bonding and reinforcement efficiency. To improve the utilization rate of fibers and the bonding strength between the textile and the concrete matrix, an arrangement along the diagonal of the grids was proposed in this paper. The flexural properties of basalt TRC plates with orthogonal grids (OG-BTRC) and plates with nonorthogonal grids (NOG-BTRC) with different grid angles and grid sizes with different laying methods, namely, a side layout (SL) and diagonal layout (DL), were studied through four-point bending tests. A comparative analysis was carried out with an ABAQUS simulation and the test results. The results showed that with a decrease in the grid angle, the BTRC specimens gradually showed a failure mode of multiple cracks, and most of the cracks appeared in the pure bending area; as the grid angle decreased, the BTRC specimens exhibited excellent flexural bearing capacity, good ductility, and high toughness. The total number of cracks on the specimen increased when it failed, while the spacing of the cracks decreased, and the fracture morphology appeared as fine and uniform features. The toughness of the specimen with a small grid angle using the DL laying method was greater than that using the SL laying method. The software simulation value matched the test data well, which proved that the test result was reliable.

## 1. Introduction

Textile-reinforced concrete (TRC) has been studied for decades as a new building material [1]. However, the textile in traditional TRC structures is composed of warp and weft fiber bundles. The failure of TRC composites is usually due to a debonding failure of the stressed warp fiber bundles. Due to the slippage between the weft fiber bundle and the warp fiber bundle during the cracking process of the composite, the reinforcement effect of textile on concrete materials is limited to a certain extent. TRC made of most types of fabric reinforcements that contribute to increased strength have been studied. Furthermore, the deformation behavior of TRC has been analyzed and was demonstrated to be advantageous under higher residual bearing capacity, which can maintain structural integrity [2]. Aksoylu studied various properties of CFRP- and GFRP-reinforced composites and obtained many important achievements [3,4,5,6,7,8,9,10,11]. Yin and Xu enhanced the flexural properties of TRC by sanding the fabric’s surface and incorporating polypropylene fibers into the concrete [12]. The sudden rupture of concrete can be overcome by combining textiles and steel bars with high ductility [13]. Wang studied the enhancement effect of textile on the flexural properties of self-stressed concrete [14,15]. Peled found a greater flexural bearing capacity in U-shaped fabric TRC beams without delamination than in steel-cage rebar TRC beams [16]. Kurban observed that the flexural strength and toughness increased with the use of hybrid yarn in TRC structures [17]. Kim found an accelerated construction method to strengthen RC slab-type elements in flexure by using precast lap-spliced TRC panels to increase the initial cracking load and the stiffness of the strengthened specimens [18]. A recent study found that textile-reinforced-polymer concrete significantly improved flexural capacity, and provided superior ductility and substantial plasticity compared with TRC [19]. The existing research indicates that the bonding performance at the interface between TRC permanent formwork and cast-in-place concrete is better than that between traditional formwork and cast-in-place concrete [20,21,22,23,24,25,26,27].

In the process of concrete cracking, cracks extend from the edge to the inside. A certain angle exists between the warp–weft textile in the cracked area and the crack, which is not the optimal arrangement. Mobasher et al. showed that at a very small orientation of 6°, the composite exhibited higher tensile-load-carrying capacity than a composite tested in the fiber orientation, that is, at 0° [28]. Therefore, a basalt TRC with nonorthogonal grids (NOG-BTRC) and a diagonal layout (DL) laying method are proposed in this paper. In this paper, through four-point bending tests of NOG-BTRC plates and OG-BTRC plates, the effects of the NOG angle, textile laying method, and grid size on the ultimate bending stress, ultimate deflection, toughness, number of cracks, and failure modes of composites were analyzed. In combination with finite element software, the experimental data were analyzed and verified.

## 2. Experiment Program

### 2.1. Material Properties

#### 2.1.1. Matrix

To ensure the strength of the composite material, the concrete matrix mix ratio was adjusted.

Table 1 shows the mix ratio of the concrete matrix. P·O 42.5 ordinary Portland cement was used in this experiment. Coarse aggregates whose nominal particle size was 4.75–10 mm and whose fine aggregates had an average fineness modulus of 2.5, which is small enough but retains sufficient strength, were used in this experiment. Sika’s third-generation polycarboxylate concrete superplasticizer was used to improve the working performance of the mixture. Pure water was used as the mixing water.

#### 2.1.2. Basalt Textile

In the experiment, continuous basalt fiber, a new high-performance inorganic fiber material made of basalt ore after high-temperature melting and drawing, was selected as the fiber material. Its main components are SiO_2_, Al_2_O_3_, CaO, FeO, Fe_2_O_3_, and TiO_2_. The filament surface is easily wetted and exhibits high alkaline resistance. Therefore, fiber roving is suitable for cement-based composite applications.

The grid angles were divided into four: 30°, 45°, 60°, and 90°. The grid sizes were divided into two: 20 mm × 20 mm and 40 mm × 40 mm. To ensure structural integrity, the textiles were impregnated with a special binder mixed with epoxy resin, curing agent, and absolute ethanol, wherein the volume ratio of epoxy resin to curing agent was 5:1. The epoxy resin penetrated into the fiber bundle, and after the epoxy resin and the curing agent were fully reacted, the synergistic force capacity of the textile and the concrete matrix could be effectively improved. The mechanical properties of the impregnated fiber bundles are shown in Table 2.

**Figure 1:**
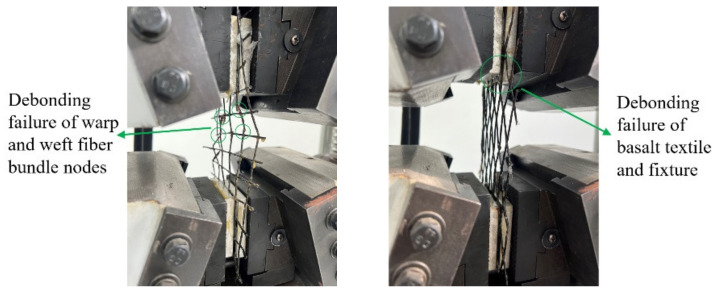
Deformation performance test of epoxy-impregnated basalt textile under uniaxial tensile test. Debonding failure of warp and weft fiber bundle nodes (**left**); debonding failure of basalt textile and fixture (**right**).

Table 3 shows the deformation capacity of tested pieces according to specifications [29]. The failure modes of tensile tests are shown in Figure 1.

### 2.2. Test Specimens

**Figure 2:**
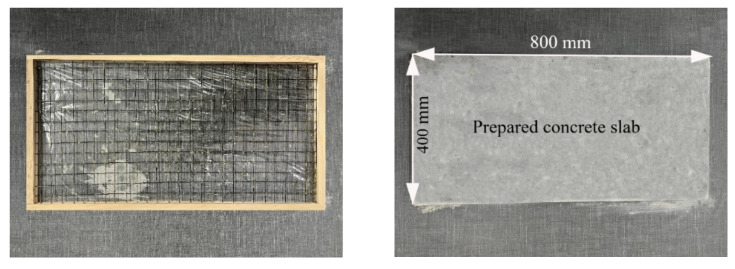
Wooden mold (**left**); cured concrete slab (**right**).

In order to reduce the influence of an uneven distribution of concrete strength, a large size of 800 mm × 400 mm × 20 mm thin-plate specimens was used in this experiment, as shown in Figure 2. A removable wooden mold was needed to hold and fix the textile. The 400 mm wide side used a 20 mm thick board for fixing, and the 800 mm long side used a 10 mm thick board for fixing. The textile was laid on it, and then it was fixed on the 100 mm thick board on the 800 mm long side. Freshly mixed concrete was poured, the sample was lightly vibrated, and the surface was smoothed. The lower surface was covered with a layer of plastic wrap to prevent slurry leakage, and the top surface was covered with plastic wrap to prevent water loss and evaporation.

**Figure 3:**
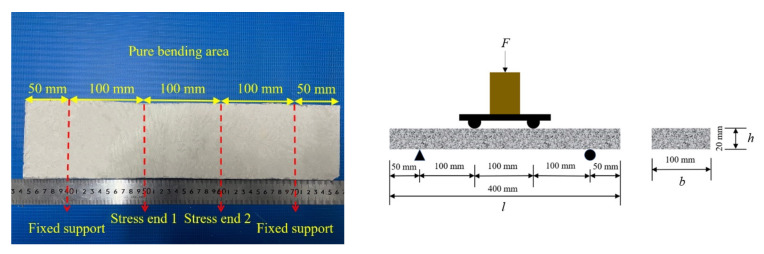
BTRC plate specimen. Division of four-point bending test area of test specimen (**left**); schematic diagram of four-point bending test (**right**).

The prepared specimens were placed at room temperature for 24 h and then removed from the mold. Next, the specimens were cured in a standard curing box for 28 days and then taken out. The cured 800 mm × 400 mm × 20 mm thin-plate specimens were processed and divided into 400 mm × 100 mm × 20 mm sections required for the four-point bending tests, as shown in Figure 3. The plate was divided into 8 small slabs lengthwise, in which basalt fabric was not laid on the left or right sides as the control group of the plain concrete thin slab. The middle six sheet samples were a group, and a layer of basalt fabric was laid at a height of 10 mm. The test group numbers are shown in Table 4.

### 2.3. Four-Point Bending Test

**Figure 4:**
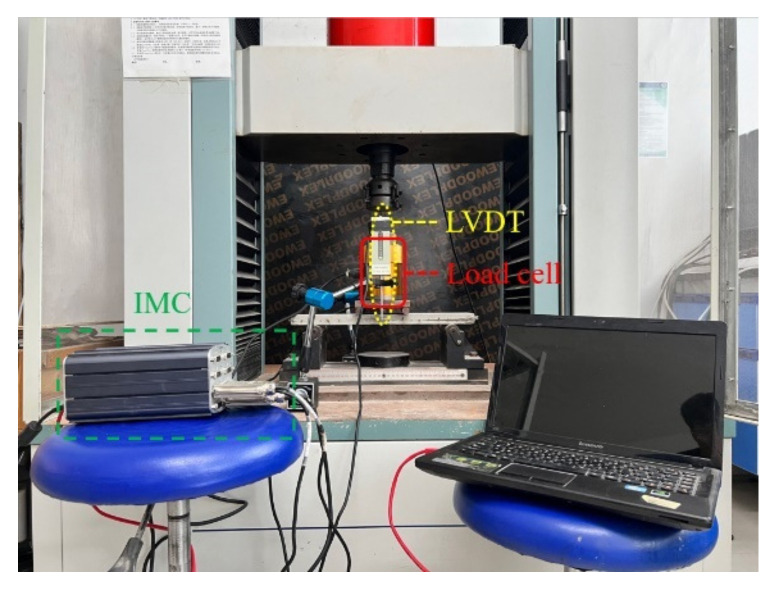
Four-point bending test of BTRC plate.

The loading method of the four-point bending test of the thin plate is shown in Figure 4, in which the plate span as 300 mm, and the shear span was 100 mm. The load-deflection full curve was collected by the IMC dynamic system, in which the load was measured by the load cell, and the mid-span deflection was measured by the LVDTs.

The specification points out that the load F and the mid-span deflection ω are related to the size of the bending specimen. To better compare the bending performance of different specimens, the test results were expressed in the form of bending stress at midspan deflection curves.

The calculation formula of bending stress is shown in the following formula [30]:

$$\sigma =\frac{Fl}{bh^{2}}$$

where *σ* denotes the bending stress, *F* denotes the bending load, *l* denotes the span of the specimen, *b* denotes the width of the specimen, and *h* denotes the thickness of the specimen.

Toughness, as an important parameter used to characterize the properties of fiber-reinforced cement-based composites, is used to describe the ability of composites to absorb energy during load failure. With the improvement of the toughness of the composite material, its various mechanical properties will be greatly enhanced. To describe the flexural resistance of the material in the four-point bending test, the toughness was compared using the calculation formula in the specification ASTM/C 1609, which is as follows [31]:

$$U_{T}=\int Pdf=\frac{bh^{2}}{l}\int \sigma df$$

The BTRC specimens had three failure modes in the test: (1) tensile failure caused by the breaking of weft fiber bundles in the textile; (2) debonding failure caused by slippage between the textile and the concrete matrix; (3) shear failure of the composite material along the oblique section. The specimens with a grid size of 20 mm × 20 mm and the SL laying method mostly suffered tensile failure due to breaking of the weft fiber bundles; most of the specimens with a grid size of 20 mm × 20 mm and the DL laying method suffered debonding failure; and the specimens with a grid size of 40 mm × 40 mm all suffered tensile failure due to breaking of the weft fiber bundles. When statistical test data were obtained, the maximum and minimum values in the data were discarded, and the valid data within the interval were taken as the reference standard.

## 3. Experimental Results

Table 5 summarizes the results of all bending tests.

### 3.1. Influence of Different Laying Methods on the Bending Properties of BTRC Plates

**Figure 5:**
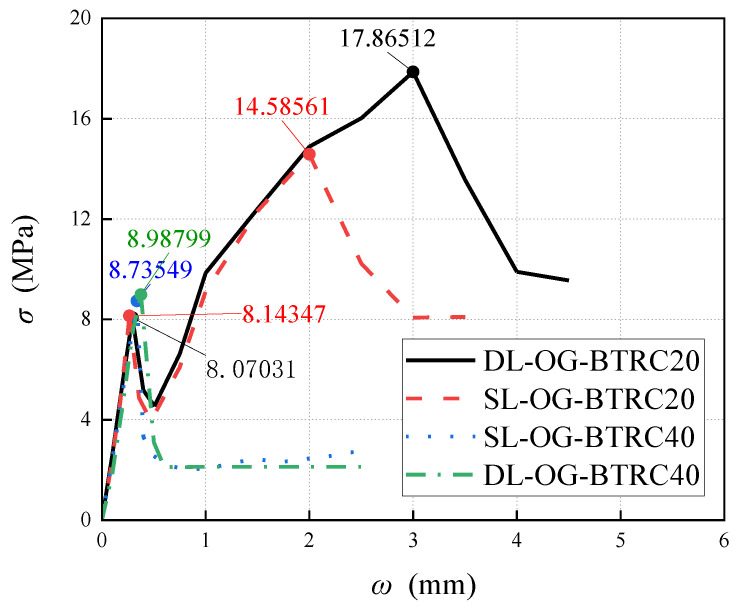
Bending stress–midspan deflection curve of BTRC plates with different laying methods.

In Figure 5, curves SL-OG-BTRC20 and DL-OG-BTRC20 were taken as one group, and curves SL-OG-BTRC40 and DL-OG-BTRC40 were taken as the other group. The comparison was obtained for the BTRC plates with different laying methods. The effect of bending stress–midspan deflection curves and the effect of different laying methods on the bending stress are shown in the figure, and the test results with different laying methods are summarized in Table 5.

**Figure 6:**
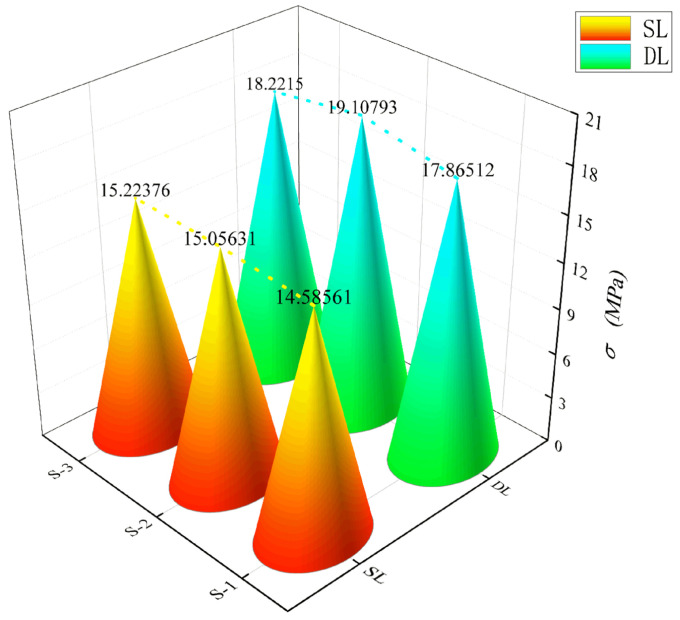
Ultimate bending stress of BTRC sheet using different laying methods.

Through the comparison of Figure 5 and Figure 6, it was found that, at the initial stage of loading of the BTRC plate, the curve showed a linear upward trend. At that time, the specimen was in the elastic stage, the textile and the concrete matrix were in the coworking stage, and the midspan deflection of the specimen increased linearly with the load. After the curve reached the peak point, the BTRC plates with different laying methods had different effects on the bending resistance of the BTRC plates due to the different grid sizes. The bending stress of the specimen with a grid size of 40 mm × 40 mm decreased rapidly after the peak value, and no secondary rise stage occurred. At that time, the toughness of the specimen under the DL laying method had improved by 15.06% compared with the SL laying method. The bending stress of the specimen with a size of 20 mm × 20 mm first decreased rapidly and then increased slowly after the peak value. At that time, the toughness of the specimen in the DL laying method was reduced by 44.91% compared with the SL laying method.

### 3.2. Effects of Different Grid Angles on the Bending Properties of BTRC Plates

**Figure 7:**
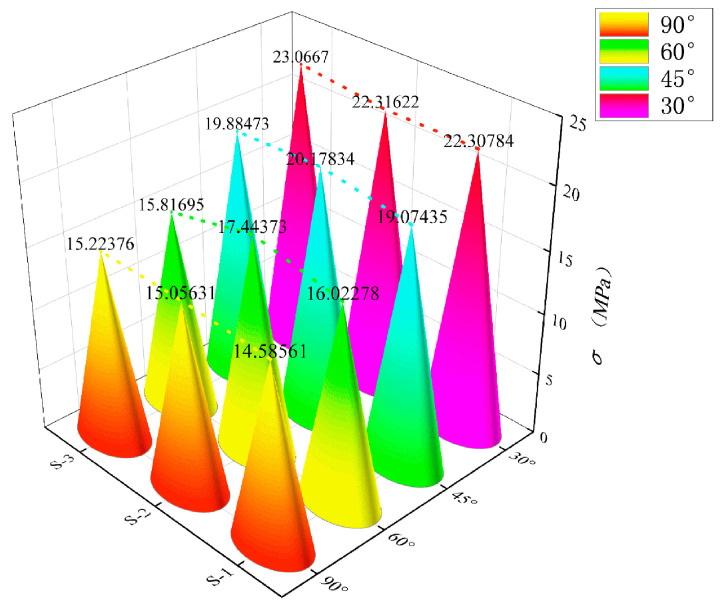
Ultimate bending stress of BTRC plates with different grid angles using the DL laying method.

Figure 7 shows the effect of different grid angles on the bending stress–midspan deflection curves of BTRC plates. The graph and data from Table 5 showed that the change of the grid angle hardly affected the cracking stress and cracking deflection of the specimen. However, the ultimate bending stress and ultimate midspan deflection of the specimen increased with a decrease in the grid angle, and the total number of cracks in the pure bending section also increased when the specimen failed. Among the specimens with a grid size of 40 mm × 40 mm, the ultimate stress of DL-NOG60-BTRC40 was 14.52% higher than that of DL-OG-BTRC40, the toughness increased by 5.09%, and only one main crack appeared when the specimen failed. The ultimate stress of DL-NOG45-BTRC40 was 17.90% greater than that of DL-OG-BTRC40; the toughness increased by 15.36%; and only one main crack appeared when the specimen failed. The ultimate stress and ultimate deflection of DL-NOG30-BTRC40 increased by 28.10% and 11.40%, respectively, compared with DL-OG-BTRC40, the toughness improved by 42.27%, and only one main crack appeared when the specimen was damaged. Among the specimens with a grid size of 20 mm × 20 mm, the ultimate stress and ultimate deflection of DL-NOG60-BTRC20 increased by 7.67% and 22.95%, respectively, compared with DL-OG-BTRC20, and the toughness increased by 32.37%. Compared with DL-OG-BTRC20, the ultimate stress and ultimate deflection of DL-NOG45-BTRC20 increased by 23.89% and 44.78%, respectively, the toughness increased by 79.39%, and one to two main cracks appeared when the specimen failed. The ultimate stress and ultimate deflection of DL-NOG30-BTRC20 increased by 44.53% and 199.52%, respectively, compared with DL-OG-BTRC20, the toughness increased by 333.26%, and two to four main cracks appeared when the specimen failed.

### 3.3. Effects of Different Grid Sizes on the Bending Properties of BTRC Plates

**Figure 8:**
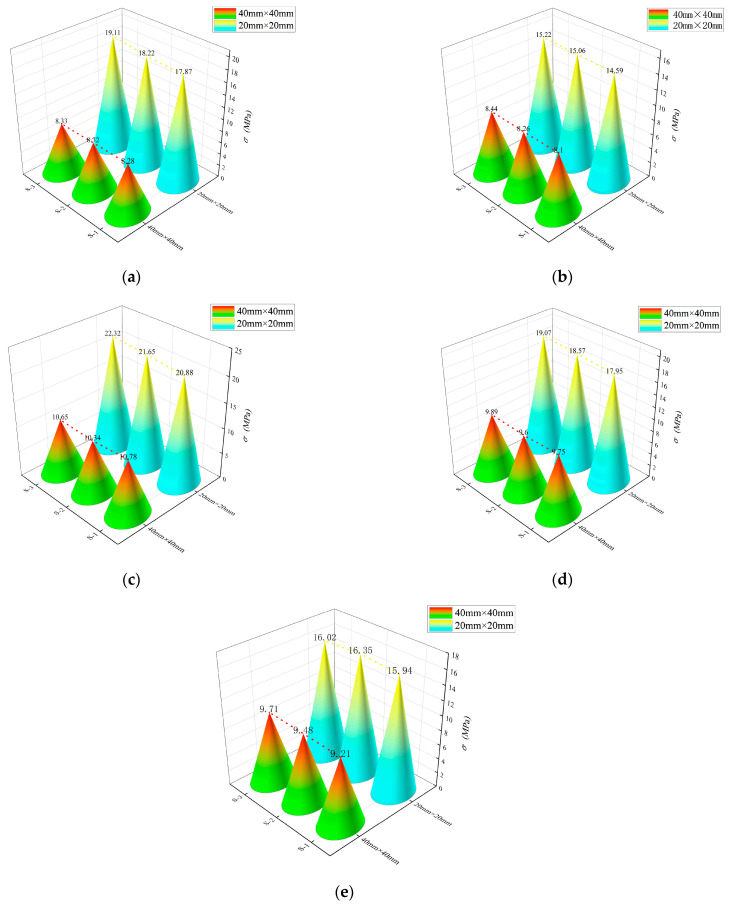
Ultimate bending stress of BTRC plates with different grid sizes. (**a**) SL-OG-BTRC20(40); (**b**) DL-OG-BTRC20(40); (**c**) DL-NOG-BTRC20(40); (**d**) DL-NOG-BTRC20(40); (**e**) DL-NOG60-BTRC20(40).

Figure 8 shows the effect of different grid sizes on the bending stress–midspan deflection curves of BTRC sheets.

The graph and data from Table 5 clearly show that the change in grid size hardly affected the cracking stress and cracking deflection of the specimen. However, as the grid size decreased, the ultimate bending stress and ultimate deflection increased greatly. With regard to the SL laying method, the ultimate stress and ultimate deflection of BTRC20 increased by 121.42% and 828.28%, respectively, compared with BTRC40, and the toughness increased by 1955.85%. With regard to the DL laying method, the ultimate stress and ultimate deflection of BTRC20 increased by 80.93% and 446.49%, respectively, compared with BTRC40, and the toughness increased by 884.31%. When the grid angle was 60°, the ultimate stress and ultimate deflection of BTRC20 increased by 70.11% and 629.52%, respectively, compared with BTRC40, and the toughness increased by 1139.82%. When the grid angle was 45°, the ultimate stress and ultimate deflection of BTRC20 increased by 90.12% and 705.36%, respectively, compared with BTRC40, and the toughness increased by 1430.70%. When the grid angle was 30°, the ultimate stress and ultimate deflection of BTRC20 increased by 104.12% and 1369.29%, respectively, compared with BTRC40, and the toughness increased by 2897.53%.

### 3.4. Analysis of Failure Modes and Constitutive Relationship of Four-Point Bending Tests

**Figure 9:**
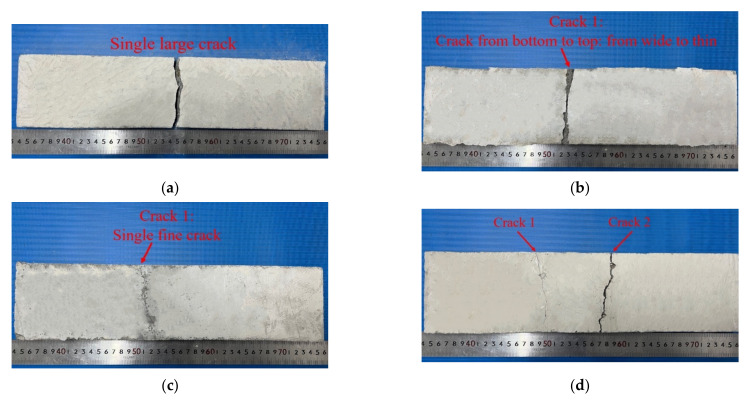

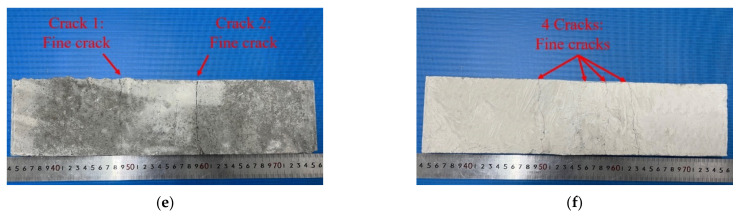
Four-point bending test failure modes of BTRC plates. (**a**) PC; (**b**) BTRC40; (**c**) SL-OG-BTRC20 DL-OG-BTRC20 DL-NOG60-BTRC20; (**d**) DL-NOG45-BTRC20; (**e**) DL-NOG30-BTRC20; (**f**) DL-NOG30-BTRC20.

Concrete is a brittle material, the failure process of PC specimens is short, and no obvious warning is given before a failure: this is known as a typical brittle failure. Only one crack appeared at the time of failure and continued to develop to the upper part of the specimen as the load increased, until the specimen was finally destroyed; the 40 mm × 40 mm basalt textile had a poor effect on enhancing the flexural performance of concrete, and only one wide crack appeared. As the load increased, the crack width increased continuously, and damage occurred as the fiber bundles between the cracks were pulled off. The concrete specimens embedded with 20 mm × 20 mm basalt textile showed obvious strain-hardening behavior, and the failure mode changed from brittle failure to ductile failure. A main crack was generated from bottom to top. When the crack reached the textile layer, it stopped extending upward and continued to generate fine microcracks around the main crack. When the intermediate textile slipped with the concrete matrix, the crack quickly extended upward from the middle layer until it was damaged. When DL-NOG45-BTRC20 failed, a crack first occurred near one of the loading points and stopped extending when it developed to the middle layer. At that time, a second crack occurred near the other loading point as the load increased continuously. As the second crack reached the middle layer, the previous crack continued to extend upwards, damaging the concrete. At that time, two cracks occurred in the specimen, one of which ran through the cross-section. DL-NOG30-BTRC20 had a large deformation when it was greatly deformed with the appearance of two to four cracks. First, two main cracks were generated near the two loading points, which gradually cracked from the bottom to the top, but they stopped when they reached the middle layer, and then secondary cracks appeared in the pure bending section, that is, between the two main cracks. With the increase in the number of secondary cracks, the bearing capacity of the specimen also gradually weakened, and the two main cracks penetrated the middle layer upwards, damaging the material. The textile played a good role in constraining the development of cracks in the concrete matrix, as shown in Figure 9e,f.

**Figure 10:**
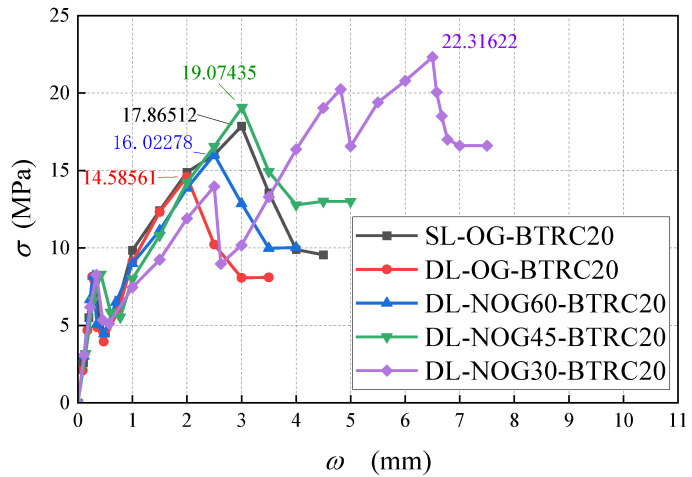
Bending stress–midspan deflection curve of BTRC20.

Table 5 and Figure 10 show that the 20 mm × 20 mm basalt textile had a significant effect on enhancing the flexural performance of concrete. The specimen could be divided into three parts: the bottom layer and the top layer were plain concrete parts, and the middle layer was the composite material part. The lower layer was plain concrete, which was why the cracking load and cracking strain of the BTRC plates were not very different from those of the PC plates. However, as the load increased, the cracks developed to the composite layer, and the load limit and deflection limit increased. The ultimate load and ultimate deflection of DL-OG-BTRC20 reduced by 23.66% and 33.78%, respectively, compared with SL-OG-BTRC20. The ultimate load and ultimate deflection of DL-NOG60-BTRC20 reduced by 8.69% and 18.65%, respectively, compared with SL-OG-BTRC20. The ultimate load and the ultimate deflection of DL-NOG45-BTRC20 were slightly higher than those of SL-OG-BTRC20. The ultimate load and ultimate deflection of DL-NOG-30-BTRC20 increased by 16.75% and 35.14%, respectively, compared with SL-OG-BTRC20. With the DL laying method, as the gird angle decreased, the bonding strength between the textile and the concrete matrix greatly improved, thereby increasing the ultimate load and ultimate slip of the composite material.

**Figure 11:**
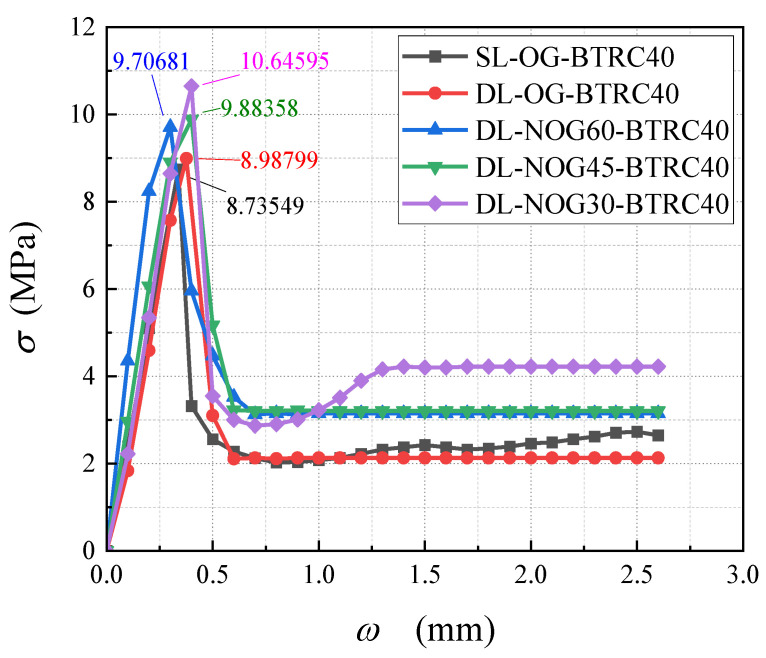
Bending stress–midspan deflection curve of BTRC40.

Table 5 and Figure 11 show that the 40 mm × 40 mm basalt textile had no obvious effect on the flexural performance enhancement of concrete. DL-OG-BTRC40, DL-NOG60-BTRC40, and DL-NOG45-BTRC40 cracked after the underlying plain concrete cracked. With the rapid expansion to the composite material layer and the low bonding strength between the 40 mm × 40 mm textile and the concrete, the composite material layer slid after being stressed, and the cracks continued to expand rapidly to the upper layer, thus, failing.

## 4. Analysis of Numerical Model

This paper used ABAQUS finite element software to run a simulation and verify the accuracy of the tests.

**Figure 12:**
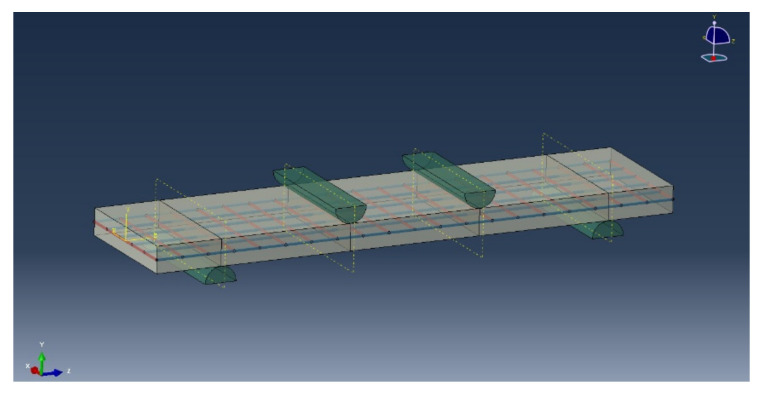
Assembly completed drawing of SL-OG-BTRC20.

First of all, during the period of creating parts, 3D, deformable, solid, and extrusion were chosen for the concrete part; 3D, deformable, wire, and extrusion were chosen for the fiber part; and 3D, analytical rigid, and extruded shell were chosen for the support part. Material properties were then given. For the concrete part, density, elastic, concrete damaged plasticity and concrete tension damage were selected. For the fiber part, density, elastic and plastic were selected. A solid, homogeneous section was chosen for the concrete and a truss was chosen for the fiber. Next, the assembly process was performed, as shown in Figure 12. After the assembly, the loading step was defined as dynamic and explicit. The next step was to define the interaction, with contact for supports and concrete and an embedded region for fiber bundles. The lower ends were fixed, and the upper two supports were applied with a downward displacement. As the height of the specimen was 20 mm and the textile was laid in the middle layer, the mesh size was defined as 10 mm × 10 mm, which caused the composite to be clearly divided into layers. Finally, the last step was to run the job operation.

**Figure 13:**
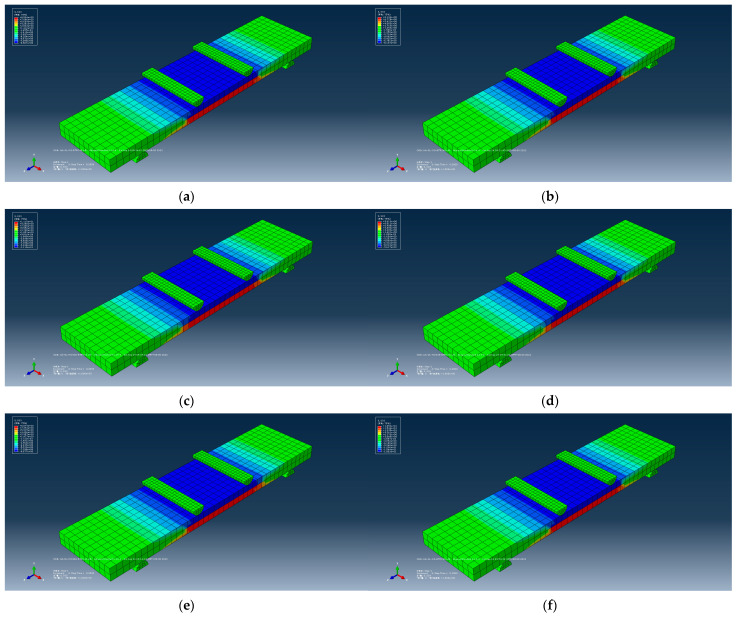

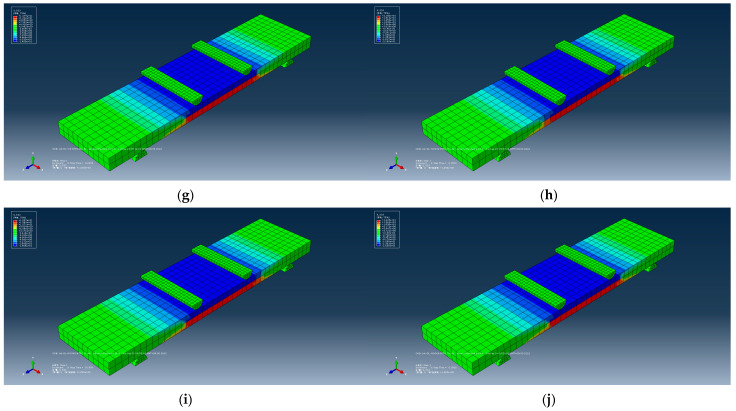
S33 Mises stress clouds of BTRC specimens. (**a**) SL-OG-BTRC40; (**b**) DL-OG-BTRC40; (**c**) DL-NOG30-BTRC40; (**d**) DL-NOG45-BTRC40; (**e**) DL-NOG60-BTRC40; (**f**) SL-OG-BTRC20; (**g**) DL-OG-BTRC20; (**h**) DL-NOG30-BTRC20; (**i**) DL-NOG45-BTRC20; (**j**) DL-NOG60-BTRC40.

The running results are shown in Figure 13. The red part in the figure represents the tensile area of the composite, and the blue part represents the compression area of the composite. These pictures characterize the stress value S33 in the z direction.

Table 6 shows the error analysis between the simulated value and the experimental value, and the results have good agreement.

## 5. Conclusions

In this paper, a new textile laying method and grid angles were studied. Four-point bending tests of BTRC plates with different grid angles, laying methods, and grid sizes were used. All data on ultimate bending strength and deformation capacity were analyzed. Different types of cracking modes were analyzed, and finite element software was used to verify the reliability of the test data. The conclusions drawn are as follows:

(1) As the grid angle decreased, the BTRC specimen exhibited excellent flexural bearing capacity, good ductility, and high toughness, and the total number of cracks in the pure bending section of the specimen increased, the crack spacing decreased, and the crack morphology appeared as fine and uniform features.
(2) The toughness of the specimen with a small grid angle and a DL laying method was greater than that with a SL laying method.
(3) As the grid size decreased, the ultimate bending stress and ultimate deflection of the specimen increased greatly. The change in grid size hardly affected the cracking stress and cracking deflection of the specimen.
(4) The simulation results verified the reinforcing effect of the textile on concrete and the retarding effect on cracks in the tests. The simulation value obtained by the finite element software had a high degree of agreement with the test data results. Thus, the four-point bending test results were reliable.

## Figures and Tables

**Table 1:** Concrete mix ratio kg/m^3^.

Water	Cement	Sand	Limestone Gravel	Superplasticizer
206	458	961	641	1.87

**Table 2:** Mechanical performance parameters of epoxy-impregnated fiber bundle.

Fiber Type	Number of Filaments Per Bundle/k	Tensile Strength/MPa	Fiber Bundle Density g/cm^3^	Elastic Modulus/GPa	Fiber Bundle Linear Density g/1000 m
Basalt fiber	3.6	2100	2.63	98.7	2400

**Table 3:** Mechanical properties of epoxy-impregnated textile.

Specimen Number	Destruction Form	Mean Ultimate Load/kN	Mean Debonding Load/kN	Deformation
BT20-SL90	Debonding of warp and weft fiber bundles	0.788	0.330	0.2675%
BT40-SL90	Debonding of warp and weft fiber bundles	0.303	0.166	0.2933%
BT20-DL90	Debonding of basalt textile and fixture	0. 765	0.328	0.2665%
BT40-DL90	Debonding of basalt textile and fixture	0.199	0.053	0.6250%
BT20-DL60	Debonding of basalt textile and fixture	0.923	0.412	0.1732%
BT40-DL60	Debonding of basalt textile and fixture	0.400	0.201	0.4156%
BT20-DL45	Debonding of basalt textile and fixture	1.055	0.443	0.1283%
BT40-DL45	Debonding of basalt textile and fixture	0.654	0.296	0.3030%
BT20-DL30	Debonding of basalt textile and fixture	1.150	0.490	0.0855%
BT40-DL30	Debonding of basalt textile and fixture	0.923	0.412	0.2083%

Note: BT20—basalt textile with 20 mm × 20 mm grids; BT40—basalt textile with 40 mm × 40 mm grids; SL90—side layout arrangement with orthogonal grid angle; DL90—diagonal layout arrangement with orthogonal grid angle; DL60—diagonal layout arrangement with nonorthogonal grid angle of 60°; DL45—diagonal layout arrangement with nonorthogonal grid angle of 45°; DL30—diagonal layout arrangement with nonorthogonal grid angle of 30°.

**Table 4:** Specimen grouping.

Grid Size	Grid Type	Grid Angle	Laying Method	Group
0	0	0	0	PC
20 mm × 20 mm	OG	90°	SL	SL-OG-BTRC20
			DL	DL-OG-BTRC20
	NOG	30°	DL	DL-NOG30-BTRC20
		45°	DL	DL-NOG45-BTRC20
		60°	DL	DL-NOG60-BTRC20
40 mm × 40 mm	OG	90°	SL	SL-OG-BTRC40
			DL	DL-OG-BTRC40
	NOG	30°	DL	DL-NOG30-BTRC20
		45°	DL	DL-NOG45-BTRC20
		60°	DL	DL-NOG60-BTRC20

Note: 0 is nothing; PC is plain concrete; OG is an orthogonal grid; NOG is a nonorthogonal grid; SL is a side layout, which means laying along the grid side; DL is a diagonal layout, which means laying along the diagonal of the grid; BTRC means basalt textile-reinforced concrete.

**Table 5:** Four-point bending tests data.

Group		Cracking Stress/MPa	Cracking Deflection/mm	Ultimate Stress/MPa	Ultimate Deflection/mm	Toughness/N·mm	Total Number of Cracks in Pure Bending Section
DL-NOG30-BTRC20	S-1	8.25	0.34	20.88	5.93	12,381.84	2
	S-2	8.32	0.40	21.65	6.22	13,466.30	2
	S-3	8.26	0.34	22.32	6.51	14,530.32	4
DL-NOG45-BTRC20	S-1	8.20	0.30	17.95	2.97	5331.15	1
	S-2	8.22	0.33	18.57	3.00	5571.00	1
	S-3	8.30	0.42	19.07	3.05	5816.35	2
DL-NOG60-BTRC20	S-1	8.22	0.25	15.94	2.53	4032.82	1
	S-2	8.29	0.31	16.35	2.58	4218.30	1
	S-3	8.18	0.28	16.02	2.55	4085.10	1
DL-OG-BTRC20	S-1	8.14	0.30	14.59	2.05	2990.95	1
	S-2	8.22	0.35	15.06	2.08	3132.48	1
	S-3	8.20	0.33	15.22	2.10	3196.20	1
DL-NOG30-BTRC40	S-1	8.33	0.31	10.78	0.46	495.88	1
	S-2	8.25	0.25	10.34	0.37	382.58	1
	S-3	8.34	0.30	10.65	0.44	468.60	1
DL-NOG45-BTRC40	S-1	8.21	0.20	9.75	0.38	370.50	1
	S-2	8.33	0.25	9.60	0.35	336.00	1
	S-3	8.38	0.29	9.89	0.39	385.71	1
DL-NOG60-BTRC40	S-1	8.10	0.26	9.21	0.33	303.93	1
	S-2	8.17	0.28	9.48	0.35	331.80	1
	S-3	8.24	0.29	9.71	0.37	359.27	1
DL-OG-BTRC40	S-1	8.10	0.37	8.10	0.37	302.13	1
	S-2	8.26	0.38	8.26	0.38	315.53	1
	S-3	8.44	0.39	8.44	0.39	329.16	1

**Table 6:** Error analysis between the simulated value and the experimental value of S33.

Group	Simulated Value/MPa	Experimental Value/MPa	Relative Deviation/%
SL-OG-BTRC20	18.50	18.40	0.54
SL-OG-BTRC40	8.51	8.31	2.35
DL-OG-BTRC20	14.80	14.96	1.08
DL-OG-BTRC40	8.14	8.27	1.60
DL-NOG30-BTRC20	21.80	21.62	0.83
DL-NOG45-BTRC20	19.10	18.53	2.98
DL-NOG60-BTRC20	16.37	16.10	1.65
DL-NOG30-BTRC40	11.10	10.60	4.50
DL-NOG45-BTRC40	9.62	9.74	1.25
DL-NOG60-BTRC40	8.87	9.46	6.65

## Data Availability

The data that support the findings of this study are available from the author (Tianqi Zhang) upon reasonable request.
